# IgA Vasculitis With Nephritis Following Controlled Ovarian Stimulation and Oocyte Donation

**DOI:** 10.7759/cureus.97026

**Published:** 2025-11-17

**Authors:** James Brader, Robin Ramphul

**Affiliations:** 1 Nephrology, Frimley Park Hospital, Frimley, GBR

**Keywords:** auto-immune diseases, controlled ovarian stimulation, iga nephritis, immunoglobulin a vasculitis, kidney biopsy, oestrogen

## Abstract

A woman in her 20s developed IgA vasculitis with nephritis (IgAVN), which started four days after oocyte donation (for in-vitro fertilisation (IVF) and transfer to another recipient). Prior to oocyte retrieval, the patient received a course of controlled ovarian stimulation (COS) with menotrophin, ganirelix acetate and choriogonadotropin alfa. Four days after COS, she developed a painful purpuric rash alongside knee pain and swelling. Prednisolone was started for presumed IgA vasculitis. Soon after, she developed de novo mononeuritis multiplex and renal dysfunction characterised by haematoproteinuria and nephrotic syndrome. A kidney biopsy confirmed the diagnosis of IgAVN. Symptoms improved over time; however, there remained questions about the possible trigger. The induced sex hormone changes associated with COS may have had an immunomodulatory effect, leading to disease development.

## Introduction

IgA vasculitis (IgAV), also known as Henoch-Schönlein purpura, is a systemic vasculitis characterised by IgA1-dominant immune complex deposition [1], often presenting with a classic tetrad of palpable purpura, arthralgia, abdominal pain, and renal involvement [2]. The pathogenesis has not been fully elucidated; however, it is thought to involve both environmental triggers and abnormal immunologic responses [1].

Environmental factors such as infections, medications, or vaccinations may act as initial triggers that stimulate mucosal B-cell production of aberrantly glycosylated IgA1 [1]. This form of IgA1 is deficient in galactose, rendering it immunogenic [1].

Subsequent immune recognition of galactose-deficient IgA1 (Gd-IgA1) by anti-glycan IgG or IgA antibodies leads to the formation of circulating immune complexes [1]. These complexes deposit in the capillaries of the skin, the glomerular mesangium, the gastrointestinal tract, and the synovium, triggering an inflammatory response via activation of the complement system, typically through the alternative and lectin pathways [1]. The inflammatory response leads to leukocytoclastic vasculitis and glomerulonephritis [1]. In this case report, we will explore the potential link between controlled ovarian stimulation (COS) and the subsequent hormonal changes to this complex pathogenesis.

The terminology to describe IgA vasculitis with renal involvement can vary, sometimes termed 'IgA vasculitis with nephritis' (IgAVN) and sometimes termed 'IgA vasculitis with IgA nephropathy' (IgAV with IgAN); for the purposes of this report, we will use the former.

## Case presentation

A woman in her 20s, previously well with no significant past medical history, received a 12-day course of COS prior to oocyte donation. Ovarian follicular stimulation was initiated with Menopur (menotrophin) at a daily dose varying between 225 IU and 300 IU. She then received Fyremadel (ganirelix acetate) at a daily dose of 0.25 mg to prevent premature ovulation, followed by Ovitrelle (choriogonadotropin alfa) as a single dose of 250 mcg to promote final oocyte maturation and ovulation. Oocyte collection was performed, during which time she received the following medications perioperatively: midazolam 2.5 mg, alfentanil 1 mg, propofol 300 mg, flumazenil 100 mcg, paracetamol 1 g, codeine 60 mg, ondansetron 4 mg, and diclofenac 100 mg.

During COS, there is a balance to be struck between efficacy and the risk of complications such as ovarian hyperstimulation syndrome (OHSS). When the patient attended for oocyte collection, the clinician reported some concern for OHSS based on the features observed on the ultrasound scan.

**Figure 1:**
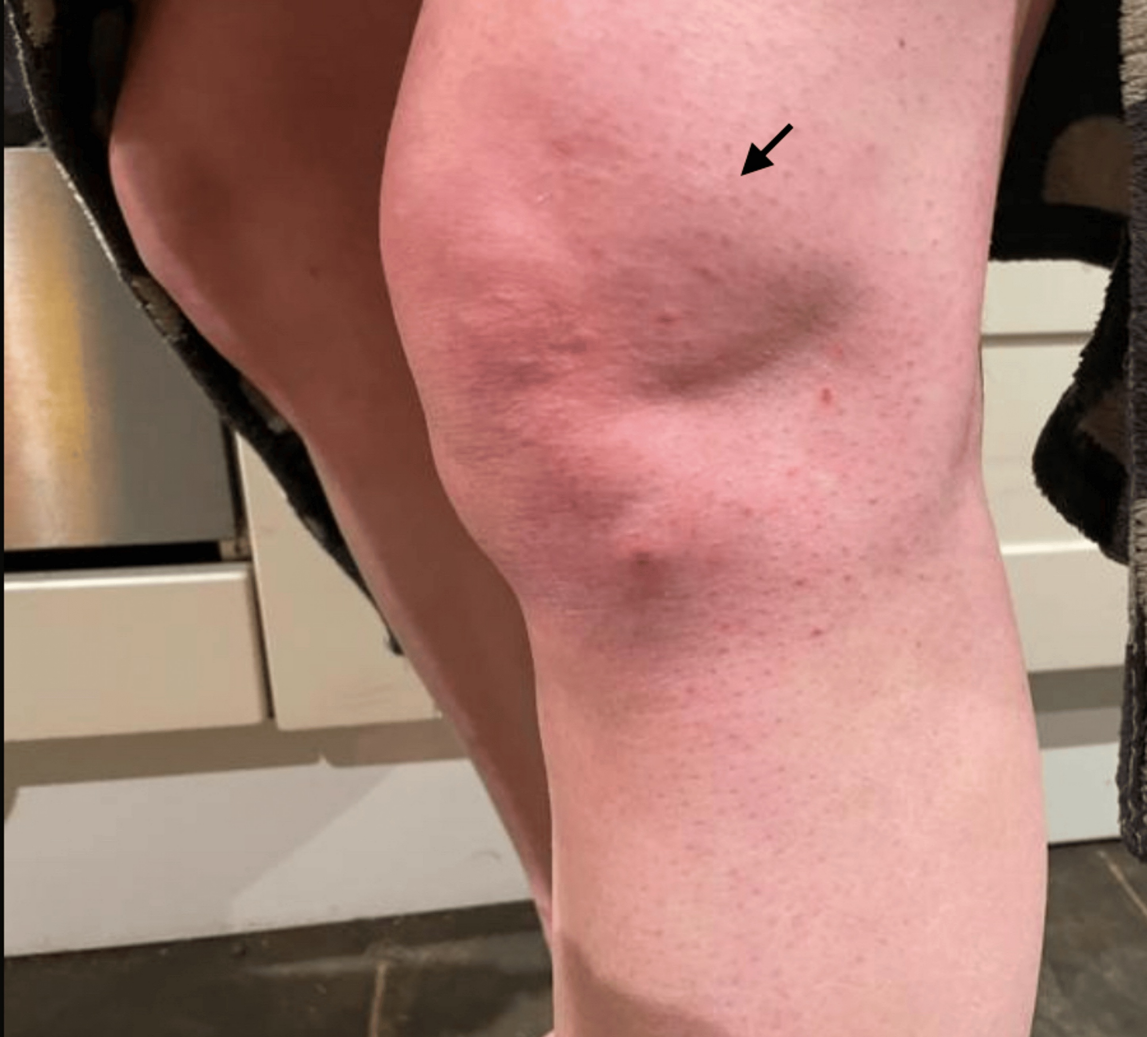
Left knee effusion. The arrow points towards the area of knee effusion.

**Figure 2:**
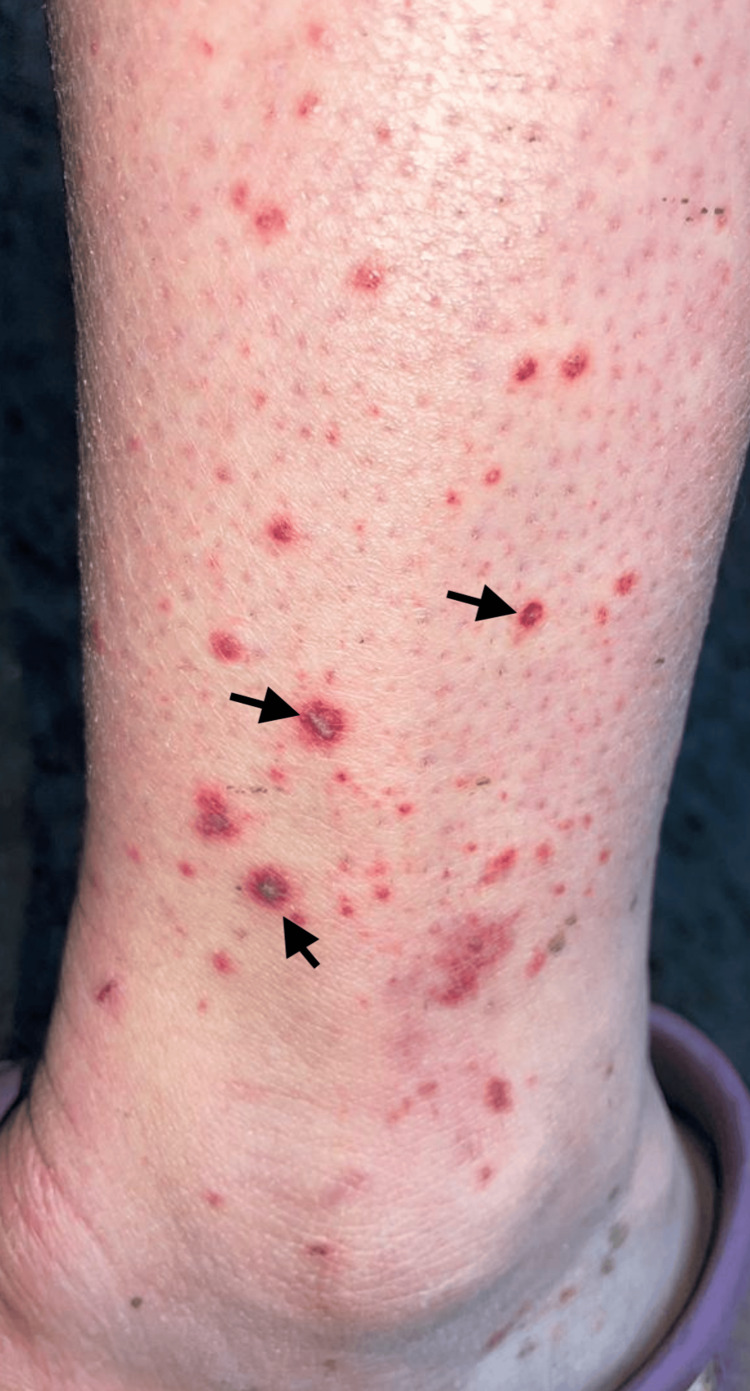
Left lower leg purpuric rash. The arrows point towards examples of purpura.

Four days after oocyte collection, the patient developed left knee pain and swelling, consistent with a left knee joint effusion (Figure 1), and a purpuric rash on the left leg (Figure 2). She presented to the emergency department. There were no preceding symptoms suggestive of an ear, nose, throat, gastrointestinal, or respiratory tract infection. The patient reported no family history of vasculitis or autoimmune disease. Initial investigations were performed (Table 1), which were in keeping with a diagnosis of IgAV.

**Table 1:** Initial investigations on first presentation. Abbreviations: eGFR, estimated glomerular filtration rate; CRP, C-reactive protein; MPO, myeloperoxidase; PR3, proteinase 3; ASO, anti-streptolysin O.

Test	Result	Reference Range
Haemoglobin	130 g/L	115–165 g/L
White cell count	9.5 × 10^9^/L	4–11 × 10^9^/L
Platelets	264 × 10^9^/L	150–450 × 10^9^/L
eGFR	>90 mL/min	>90 mL/min
Creatinine	68 µmol/L	49–90 µmol/L
CRP	24 mg/L	0–9.9 mg/L
Albumin	44 g/L	35–50 g/L
Complement C3	1.43 g/L	0.75–1.65 g/L
Complement C4	0.34 g/L	0.14–0.54 g/L
MPO antibodies	<0.3 U/mL	0–0.34 U/mL
PR3 antibodies	<0.7 U/mL	0–1.9 U/mL
Rheumatoid factor	<10.6 IU/mL	0–15 IU/mL
ASO titre	400 IU/mL (200 IU/mL repeated after two weeks)	>200 IU/mL suggests past infection
Joint fluid analysis (from left knee)	Negative for both growth and crystals, with high inflammatory cells noted	No reference range
Blood cultures	Negative for growth	No reference range
Urine dipstick	Negative for both blood and protein	No reference range
Chest X-ray	Unremarkable, with clear lung fields	No reference range

Following these initial investigations, she was started on prednisolone (60 mg daily) for presumed IgAV by the rheumatology team. In addition, she was prescribed omeprazole (20 mg daily) for gastric protection and Adcal D3 (calcium carbonate 1.5 g and 400 IU of colecalciferol) twice daily to support bone integrity whilst taking steroid therapy.

In the three weeks following the initiation of prednisolone therapy, she went on to develop further signs of vasculitis, despite ongoing treatment. She developed reduced sensation over the lateral aspect of her right foot corresponding to the L5/S1 dermatome. There was no associated weakness. Nerve conduction studies showed absent right superficial peroneal sensory nerve action potential (SNAP) and reduced amplitude of the right sural SNAP, consistent with mononeuritis multiplex. There was also an extension of the purpuric rash proximally up her left leg, along with de novo lesions on her right leg. Some of these lesions developed into blisters and ulcers.

During follow-up, she also developed persistent non-visible haematuria and proteinuria. Urinalysis revealed 3+ blood and 3+ protein. The urine protein-to-creatinine ratio rapidly rose to a peak of 1394 mg/mmol (normal range <3 mg/mmol). There was associated hypoalbuminaemia (albumin 30 g/L) and peripheral oedema corresponding to nephrotic syndrome. Despite this, eGFR remained ≥90 mL/min, and the highest recorded creatinine level was 77 µmol/L.

**Figure 3:**
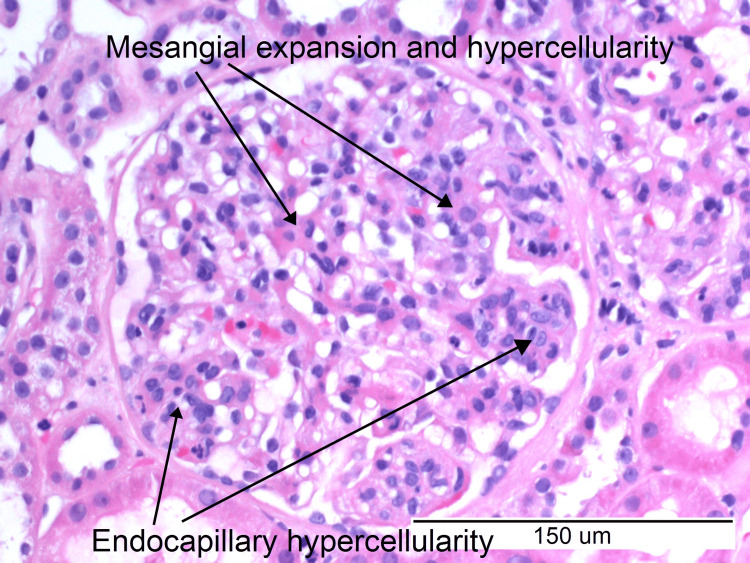
Haematoxylin and eosin stain (magnification ×20) demonstrating mesangial hypercellularity and endocapillary hypercellularity.

**Figure 4:**
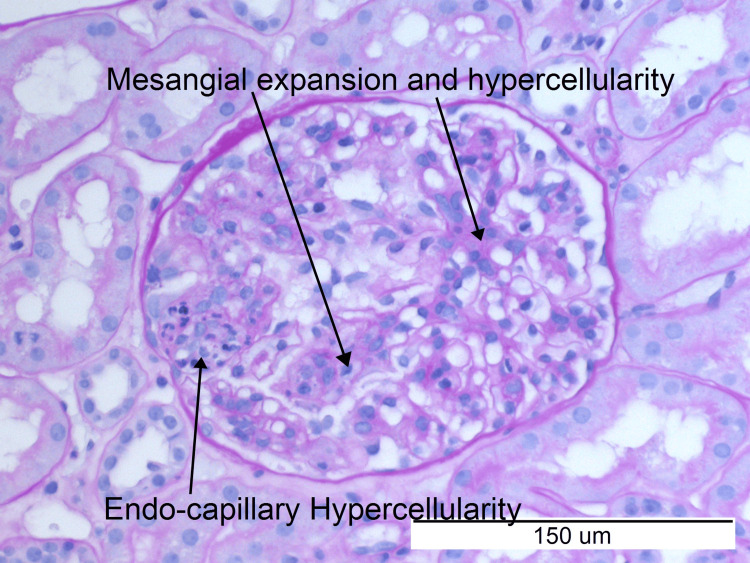
Periodic acid-Schiff stain (magnification ×20) demonstrating mesangial hypercellularity and endocapillary hypercellularity.

She was referred to the nephrology team, and under their direction, she was started on ramipril 1.25 mg for proteinuria. A kidney biopsy was performed. Histology images show mesangial expansion and hypercellularity, endocapillary hypercellularity, and a cellular crescent (Figures 3, 4).

**Figure 5:**
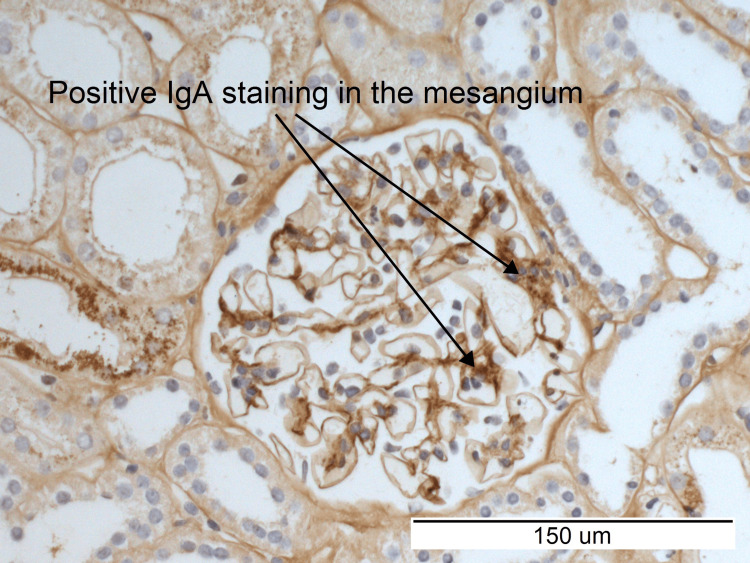
Immunohistochemistry demonstrating positive immunoglobulin A staining in the mesangium.

**Figure 6:**
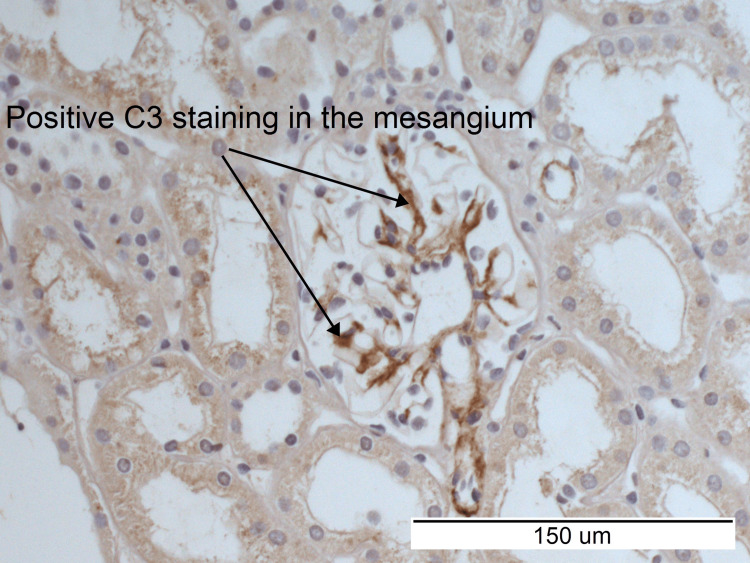
Immunohistochemistry demonstrating positive C3 staining in the mesangium. C3: Complement component 3.

Immunohistochemistry revealed positive staining for IgA and C3 (Figures 5, 6). The overall appearance of the kidney biopsy was reported by the histopathologist as consistent with early IgA nephropathy.

Follow-up continued under the direction of the nephrology team. Approximately five weeks following oocyte donation, the purpuric rash disappeared, and the sensation in her right foot improved. Knee pain and peripheral oedema have all resolved. Evidence for steroid therapy in IgAVN is controversial and used at the discretion of the physician on a case-by-case basis after considering the potential benefits and adverse effects. In this case, steroid therapy was not felt to offer any additional benefit over renin-angiotensin-aldosterone system inhibition alone. For this reason, the prednisolone dose was weaned to a complete stop in the following three months.

She was regularly reviewed in the nephrology outpatient clinic over a two-year period. Throughout this period, eGFR remained ≥ 90 mL/min, and creatinine levels remained satisfactory at baseline. However, there were continued but fluctuating levels of proteinuria, with urine protein-to-creatinine ratios ranging from 51 to 584 mg/mmol; the trend was generally a reduction in proteinuria over time. Proteinuria levels fell after an increase in ramipril dose to 2.5 mg daily, with levels at the last review of 115 mg/mmol. Attempts at optimising the dose of ramipril were hindered by missed outpatient appointments and reported symptoms suggestive of hypotension (light-headedness).

After two years, she notified the nephrology team that she was pregnant and had relocated to another area. Ramipril was discontinued, as it is contraindicated in pregnancy, and her care was transferred to the local nephrology team. She reported that there have been no further concerns raised, no relapses in symptoms and that her pregnancy was successful and uncomplicated.

## Discussion

This case describes the development of IgAVN in a previously healthy woman shortly after undergoing COS. To our knowledge, there are no prior reported cases linking COS to the onset of IgAV, IgAN or IgAVN.

Under normal conditions, reproductive hormones are regulated by the hypothalamic-pituitary-gonadal (HPG) axis. Gonadotropin-releasing hormone (GnRH) release from the hypothalamus stimulates pituitary secretion of follicle-stimulating hormone (FSH) and luteinising hormone (LH), which drive ovarian follicle development and the production of oestrogen and progesterone. Oestrogen and progesterone then exert feedback on the hypothalamus and pituitary, resulting in the cyclical hormonal fluctuations of the menstrual cycle. In this case, COS may have led to abrupt and pronounced changes in hormone levels, as there was a concern for OHSS. Menotrophin contains FSH and LH to stimulate follicle growth; ganirelix acetate (a GnRH antagonist) suppresses premature LH surges to control timing; and choriogonadotropin alfa (recombinant human chorionic gonadotropin) induces the final maturation and release of oocytes by mimicking the natural LH surge. GnRH effects are suppressed throughout COS, whilst oestrogen levels rise during menotrophin administration and peak around the time of choriogonadotropin alfa administration. Following oocyte retrieval and discontinuation of the GnRH antagonist, normal HPG axis function resumes whilst exogenous hormone levels decline and oestrogen drops rapidly during luteal phase progression.

Administration of high levels of exogenous gonadotropins, with subsequent changes in oestrogen and progesterone levels for oocyte harvesting, may affect immune homeostasis. Studies have revealed a complex and incomplete picture. Oestrogen, in particular, has been shown to modulate cytokine production and B-cell activation and increase autoantibody production by elevating levels of B-cell activating factor (BAFF) [3]. A prospective trial evaluating COS-induced immune changes in healthy, infertile women demonstrated transient alterations in B-cell subsets but found no significant changes in BAFF levels, anti-nuclear antibody titres or immunoglobulin concentrations during or after stimulation [4], suggesting that whilst COS induces temporary immune modulation, it does not significantly activate autoimmunity in immunologically normal individuals. However, this study included only a limited number of immunological parameters in a small sample of 63 infertile women and 39 healthy controls and therefore may not provide a complete picture.

Autoimmune conditions have been linked to COS in previous case reports. Graves' disease development was reported in a patient after multiple COS cycles [5]. Another case describes the development of insulin-dependent diabetes mellitus, systemic lupus erythematosus (SLE), and antiphospholipid syndrome in a patient following multiple ovulation induction cycles, raising concerns about excessive levels of oestradiol in the development of autoimmune disease in a susceptible woman [6]. In autoimmune-prone murine models, oestrogen exacerbates diseases such as SLE [7], suggesting a possible parallel in humans.

Group A Streptococcus (GAS) infection has previously been associated with the development of IgAV/IgAN. The presence of IgA-binding M proteins of GAS in the skin and kidney biopsies of multiple patients with IgAV and IgAN has been highlighted [8]; the authors suggested a pathogenetic role of these streptococcus M proteins via binding to circulatory IgA and forming complexes that can deposit in tissues, resulting in disease [8]. A different study has reframed IgAN as a tissue-specific autoimmune disease [9], reporting the presence of IgA autoantibodies directed against mesangial cell surface proteins. The authors suggest that molecular mimicry between certain oral streptococcal antigens and mesangial antigens may drive the abnormal IgA deposition seen in the mesangium. Although this study focused on *Streptococcus mutans* rather than GAS, it provides further evidence for streptococcal infection as a possible initiator of disease. The connection to COS in this case could be via a two-hit mechanism whereby GAS infection sensitises the immune system towards autoimmunity, perhaps via the M protein or the molecular mimicry theories above, which was then furthered by B-cell modulation by excessive oestrogen levels.

In the case reported here, a mildly elevated ASO titre (400 IU/mL at first presentation and 200 IU/mL at a repeat two weeks later) may be suggestive of a previous GAS infection. However, in the absence of any symptoms, this marginally elevated ASO titre is not conclusive of infection. Given that ASO titres begin to decline eight weeks after infection [10], the fall in this patient's ASO titre two weeks after initial presentation suggests any potential infection would likely have been several weeks, possibly months, prior to her presentation with IgAV. An epidemiological review of the development of IgAV in childhood considered upper respiratory tract infections as potential precipitating events if there was a close temporal relationship (less than two weeks) to the onset of the disease [11]. This review found that, in children presenting with IgAV, an ASO titre was raised in 54 of the 108 cases tested [11]; however, a recent streptococcal infection was only demonstrated with certainty in two of these 54 cases. Overall, it is considered unlikely that GAS infection contributed to the development of IgAVN in this case.

The perioperative drugs the patient received during oocyte collection have also been considered as possible triggers; however, they are felt unlikely to be contributory. A large pharmacovigilance study [12] examined the association between different medications and the development of IgAV. The most significant drug classes reported were vaccines, antibiotics, and tumour necrosis factor-α inhibitors. None of the perioperative drugs this patient received were mentioned other than non-steroidal anti-inflammatory drugs (NSAIDs). NSAIDs were reported as nine of the 178 drugs implicated in 115 cases of probable or definite IgAV. The authors noted that drug causality assessment is challenging, as many other aetiological factors can be involved. It is felt unlikely that the use of 100 mg of diclofenac or any other perioperative drug significantly contributed to disease development in this case.

## Conclusions

This case highlights a possible link between COS and the onset of IgAVN. The underlying mechanism is unclear, but it may be related to sex hormone modulation of the immune system. This case adds to several other case reports linking autoimmune disease to variations in sex hormone levels. Further studies are needed to understand the immunological consequences of COS and their relation to the underlying pathogenesis of IgAVN. Advances within this area would help clarify which triggers and factors may lead to the development of IgAVN.
